# The path to minimizing instability in developmental dysplasia of the hip: is Capsulorrhaphy a necessity or a futile habit?

**DOI:** 10.1186/s12891-021-04065-3

**Published:** 2021-02-17

**Authors:** Ramin Zargarbashi, Mohammadreza Bozorgmanesh, Behnam Panjavi, Fardis Vosoughi

**Affiliations:** 1 Department of Pediatric Orthopedics, Children’s Medical Center and school of medicine, Tehran University of Medical Sciences, Tehran, Iran; 2 Department of Orthopedic Surgery, Vali-e-Asr Hospital, Arak University of Medical Science, Arak, Iran; 3 Department of Orthopaedic and Trauma Surgery, Jalal Street, Shariati Hospital and school of medicine, Tehran University of Medical Sciences, Tehran, Iran

**Keywords:** DDH, Developmental hip dysplasia, Capsulorrhaphy, Medial joint space

## Abstract

**Background:**

To evaluate and quantify the intraoperative effect of capsulorrhaphy on the deep seating of femoral head within the acetabulum as measured by medial joint space, a surrogate measure of acetabular-head contact.

**Methods:**

In order to determine the exact effect of capsulorrhaphy, we prospectively scrutinized a consecutive sample of 18 patients with unilateral dysplastic hips aging > 18 months and followed them for a period of at least 12 months. The procedure of open reduction is described in detail. Two pediatric orthopedists carried out the operations from August 2014 to January 2019 at a tertiary pediatric hospital. Intraoperatively, AP radiographs of the pelvis were obtained before and after capsulorrhaphy. The distance between the inferomedial edge of the proximal femoral metaphysis and the lateral edge of the obturator foramen was recorded. To determine if there were differences in medial joint space due to capsulorrhaphy, a generalized linear model was run on the study sample. All patients were followed for at least 12 months to determine the rate of re-dislocation.

**Results:**

Mean age (±standard deviation) of the participants was 37.5 (±24.7) months. All cases underwent Salter osteotomy, 5 cases needed femoral shortening (27.8%) and none needed derotational osteotomy. Capsulorrhaphy lead to a statistically significant decrease in the mean medial joint space from 1.59 cm before (95% CI: 1.12–2.05) to 0.76 cm after (95% CI: 0.50–1.02) the capsulorrhaphy (*P* < 0.001). When we took the effect of age into account the corresponding figures were 1.47 (95% CI: 1.22–1.75) and 0.67 (95% CI: 0.39–0.94), respectively (P < 0.001). After follow up periods of 1 to 5.5 years, none of the patients experienced instability or re-dislocation.

**Conclusions:**

Capsulorrhaphy, independently, of age was associated with a 1-cm decrease in the mean medial hip joint space and a more deeply seated femoral head. Furthermore, this study presents a successful experience with capsulorrhaphy to prevent hip instability.

## Background

A congruent stable hip is the result of parallel geometrical development of the proximal femur and the acetabulum. In other words, it is a product of concurrent acetabular and proximal femoral growth from their corresponding growth plates [1]. Absence of appropriate contact between the acetabulum and proximal femur leads to an incongruent joint. The hip joint may not develop normally if it doesn’t reach congruity by walking age [2, 3]. Developmental dysplasia of the hip (DDH) falls somewhere in a spectrum of physical and imaging findings. Early diagnosis and treatment of DDH is critical in achieving the best possible functional outcome [3]. The acetabular changes in DDH have been described and well recognized before. Recently, we have gained better understanding of the changes in the femoral head. Patients who fail after closed treatment, need an open reduction. Despite substantial research, high-quality comparative studies continue to be lacking and the treatment of DDH is mainly based on the clinical experience of the treating surgeon [3]. This variability in opinion became evident in a 2018 survey of EPOS and POSNA members on their preferred treatment methods for DDH [4].

Principals of surgical treatment of DDH, however, always include reduction and stabilization. These principals could be applied by conservative or surgical means. Conservative measures are more effective before the age of 6 months and include a variety of techniques such as the Pavlik harness splint [5], the Tubigen hip flexion splint [6], closed reduction plus spica casting and Sommerville- Petit method of traction [7]. According to the 2018 DDH survey, in spite of marked disparity between surgeons, closed reduction is usually not favored prior to 3 months or open reduction before 6 months of age [4]. Surgical Stabilization might be required in cases with failed conservative treatment, residual dysplasia or older children with neglected DDH. Surgical stabilization is generally achieved by a reduction into a near anatomical position and perhaps a complementary capsulorrhaphy. In fact, some surgeons do not believe capsulorrhaphy to be necessary [8, 9]. Others, perform capsulorrhaphy as a routine adjuvant to open reduction [10–14]. Theoretical pros and cons have been proposed with few studies, attempting to examine the clinical relevance of the capsulorrhaphy [14]. Furthermore, correction of bony abnormalities such as excessive femoral anteversion or length and acetabular dysplasia might be indicated to prevent instability. Surgical correction of bone deformities should be aimed at securing the stabilization [12].

Treatment of DDH hinges heavily on attempting to keep the femoral head within the acetabulum (concentric reduction), thereby inducing acetabular development to contain the femoral head. Acetabular remodeling requires pressure of the well-seated femoral head against the acetabulum [15, 16]. Magnetic resonance imaging has been shown to have a role in the evaluation of joint congruency [17]. However, it is expensive with limited access in developing countries and also is not available intraoperatively [18, 19]. The width of the medial joint space on a standard AP radiograph has been widely accepted as an indicator of stability of the reduction [19]. Normal radiographic appearance, therefore, could be indirectly translated to improved outcome. Hence, clinical relevance of different treatments could be assessed on the basis of the extent to which they can restore normal radiographic appearance of the hip joint.

Within the frames of this paradigm, we hypothesized that capsulorrhaphy can improve the acetabular-head contact among patients treated surgically for DDH. Hence, the primary objective of this study is to determine if capsulorrhaphy decreases medial joint space and thereby improves acetabular-head contact. Secondary objectives are to determine if capsulorrhaphy caused limitation in hip range of motion or avascular necrosis of the femoral head. Moreover, we intend to present our experience in attempting to achieve hip stability using capsulorrhaphy as an adjuvant to open reduction among other methods.

## Methods

### Participants

The study was approved by the Institutional Review Board of Tehran University of Medical Sciences. Informed written consent was obtained from the patients’ parents or legal guardians. We prospectively reviewed a consecutive sample of 20 patients with unilateral DDH aging > 18 months. Non-idiopathic cases (e.g., due to paralytic disorders or congenital syndromes) were excluded from the study (2 cases). The remaining 18 patients were studied. Demographic data were collected using a predetermined questionnaire.

### Procedure

The procedure included a one-stage open reduction and Salter osteotomy, combined with femoral shortening or derotational osteotomy if necessary, by two surgeons, from August 2014 to January 2019 at a tertiary pediatric hospital.

### Radiologic evaluation

**Fig. 1:**
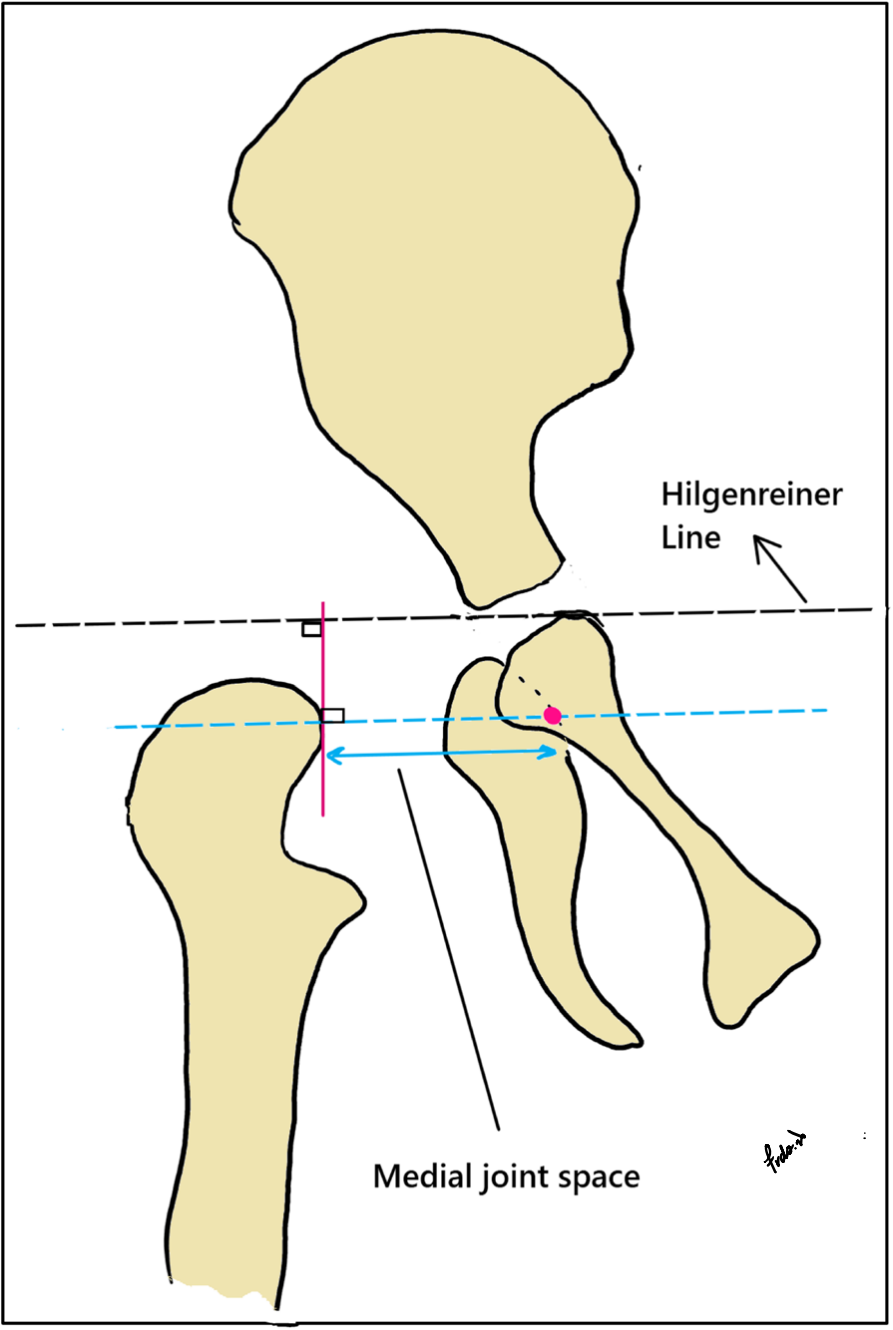
Schematic drawing of the medial hip joint space. [This illustration is the sole work of the corresponding author (F.V.), presented for the purpose of publication in the current study]

Intraoperatively, simple AP radiographs of the pelvis were obtained with the child in the supine position. Care was taken to position the child with the legs parallel and to avoid rotation of the pelvis and hips. The first radiograph was taken after the reduction of femoral head, Salter osteotomy, depleting the acetabulum of pulvinar fat and releasing the transverse ligament, just before capsulorrhaphy. The second radiograph was taken just after capsulorrhaphy. Having considered a magnification marker (surgical blade), we measured the distance between the inferomedial edge of the proximal femoral metaphysis and the lateral edge of the obturator foramen (Fig. 1). This distance was used as a surrogate measure to quantify the extent to which femoral head was deeply seated within the acetabulum.

### Surgical technique

We used a “bikini” incision extended distally for 2 cm beyond the anterior inferior iliac spine (AIIS). The external oblique muscle was shaved from the iliac crest, to allow splitting the iliac apophysis down to the bone. Blunt dissection of the tensor–Sartorius interval was performed. Tendinous part of the iliopsoas was sectioned to achieve adequate medial exposure. After gaining good exposure, capsulotomy was performed in a V-shaped manner leaving a 5-mm margin of capsule. The ligamentum teres, the fibrofatty tissue and transverse ligament were removed to provide a reducible joint. With the hip dislocated, nonabsorbable sutures were passed through the medial portion of the capsule. Next the hip was reduced, and sutures were passed through the superolateral segment of the capsule. Decision was made to perform innominate osteotomy and/or femoral shortening. Only after performing osteotomies and reducing the joint, was the joint capsule imbricated by tying the suture knots. An anteroposterior radiograph was taken by C-arm prior to and after the capsulorrhaphy. After repairing the iliac apophysis, rectus femoris, and the wound, a one-and-half hip Spica cast was applied postoperatively for 6 weeks, after which it was changed to a broomstick cast for 4 weeks.

### Statistical analysis

The data are presented as mean (SD) and frequency (%) for continuously- and categorically-distributed variables respectively. As a normal distribution was not seen, a non-parametric test seemed more appropriate. Wilcoxon signed-rank test was used to compare means. To determine if there were differences in medial joint space due to capsulorrhaphy a general linear model was run on a sample of 18 participants. All statistical analysis was performed with the use of SPSS software version 25 (IBM Corp.). *P*-value less than 0.05 was considered significant.

## Results

**Fig. 2:**
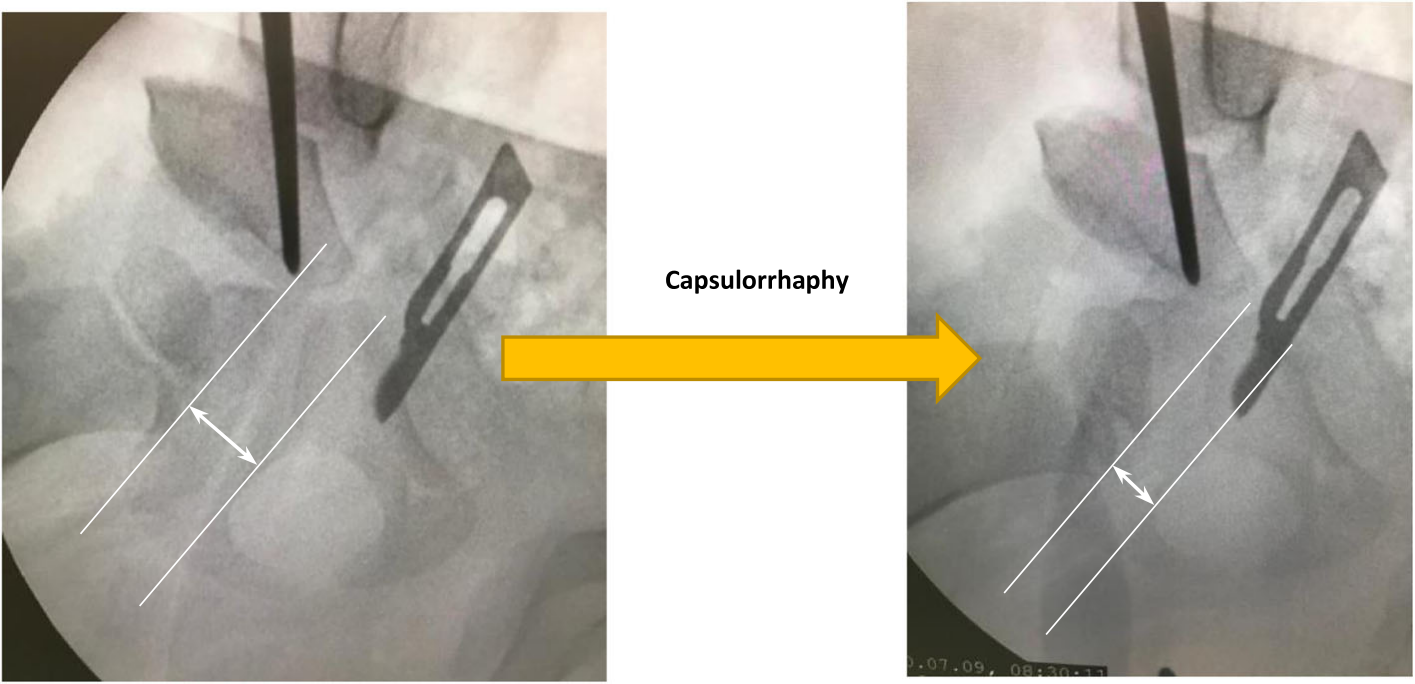
Medial joint space before (left) and after (right) capsulorrhaphy

**Fig. 3:**
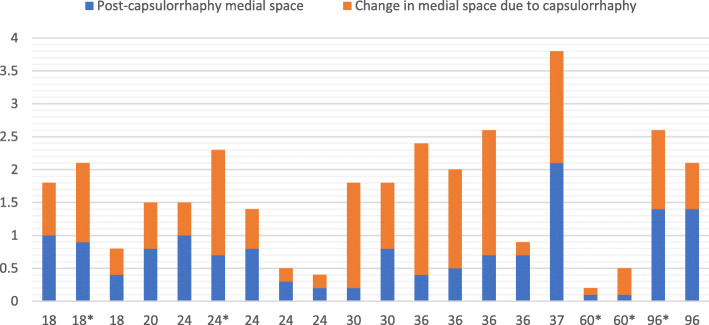
Medial Joint space for each patient before (blue+ orange columns) and after (blue columns) capsulorrhaphy. The cases are sorted by increasing age and each patient’s age is shown on the X-axis. Patients who underwent femoral shortening are marked (*). The effect of capsulorrhaphy can be seen in patients with different ages

Baseline characteristics of the participants are shown in the Table 1. Mean age (SD) was 37.5 (24.7) months (range: 18–96). 3 of them were boys (16.7%). Patients had no other underlying conditions. All the cases underwent Salter osteotomy, 5 cases (27.8%) needed femoral shortening and none needed derotational osteotomy. Medial joint space values for all patients are given in Table 2. A statistically significant decrease in mean medial joint space is seen from 1.59 just before capsulorrhaphy (95%CI: 1.12–2.05) to 0.76 (95%CI: 0.50–1.02) afterwards (*P* < 0.001). An example has been shown in Fig. 2. Moreover, medial joint space values for all patients are depicted as clustered bar charts (Fig. 3). When we took the effect of age into account, the corresponding figures were 1.47 (95%CIs: 1.22–1.75) and 0.67 (95%CIs: 0.39–0.94), respectively (P < 0.001). Also, age did not have a significant effect on the change of medial joint space (*P* = 0.26).

**Table 1:** Patient characteristics

*Patient Characteristics*	*Frequency (%)*
*Age at the time of operation (in months)*	
Mean ± S.D. (min–max)	37.5 ± 24.7 (18–96)
*Age groups (in years)*	
< 3	11 (61.1)
3–6	5 (27.8)
> 6	2 (11.1)
*Sex*	
Male	3 (16.7)
Female	15 (83.3)
*Type of surgery*	
Salter Osteotomy	18 (100)
Femoral Shortening	5 (27.8)
Femoral derotational osteotomy	None
*Mean Medial Joint Space values*	
Before Capsulorrhaphy (95% CI)	1.59 (1.12–2.05)
After Capsulorrhaphy (95% CI)	0.76 (0.50–1.02)
Before-After difference (95% CI)	0.83 (0.54–1.11)
Power analysis value	< 0.001

**Table 2:** Medial joint space values for each patient. Cases marked with a star, underwent femoral shortening

ID	Age (months)	Medial joint space (cm)	
		Before capsulorrhaphy	After capsulorrhaphy
1*	60	0.20	0.10
2*	96	2.60	1.40
3	30	1.80	0.20
4	20	1.50	0.80
5	36	2.60	0.70
6	30	1.80	0.80
7	37	3.80	2.10
8	24	1.50	1.00
9*	24	2.30	0.80
10	36	0.90	0.70
11	18	1.80	1.00
12	96	2.10	1.40
13*	60	0.50	0.10
14*	18	2.10	0.90
15	24	1.40	0.80
16	24	0.50	0.30
17	24	0.40	0.20
18	18	0.80	0.40

Mean medial joint space values were also calculated specifically among the patients who had not underwent femoral osteotomy (13 patients). Mean medial joint space of 1.61 (95%CIs: 1.05–2.16) in this group decreased to 0.80 (95%CIs: 0.48–1.12) after capsulorrhaphy, showing a mean decrease of 0.81 (95%CIs: 0.45–1.16) centimeters.

The mean duration of follow up was 2.3 years (1.1) ranging from 1 to 5.5 years. Patient evaluations in the follow up period, revealed no signs of instability, subluxation or re-dislocation after at least 12 months from surgery. All 18 patients achieved symmetric full hip range of motion with full extension. No case of avascular necrosis or osteoarthritis was seen during the follow up periods.

## Discussion

**Fig. 4:**
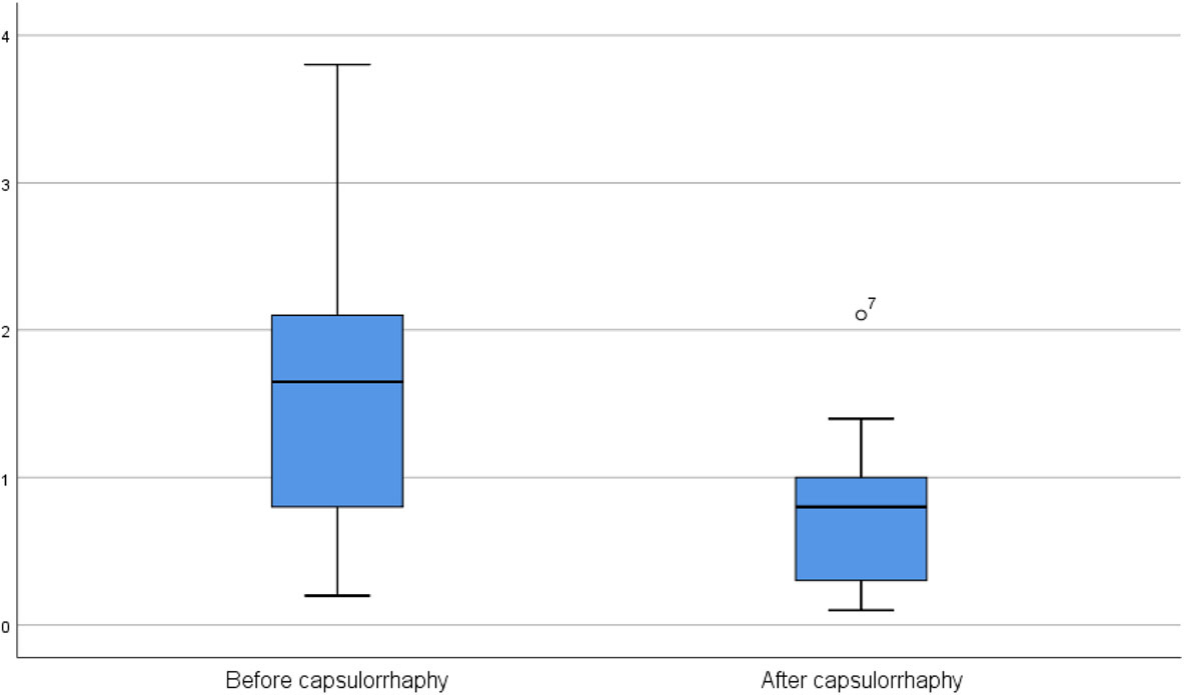
stem-and-leaf plot for mean pre-capsulorrhaphy and post-capsulorrhaphy medial joint space

Herein, this study shows that, as a part of open reduction of the DDH, capsulorrhaphy can significantly improve deep-seating of the femoral head within the acetabulum (Fig. 4).

Capsulorrhaphy has long been proposed as a part of the surgical treatment of DDH [20] and inadequate capsulorrhaphy has been reported as a factor predisposing to failure of the initial open reduction along with other factors such as inadequate inferior capsular release [21–28].

Iyetin et al. have recently introduced a modified surgical approach to the hip in which capsulorrhaphy is not required. They obtained excellent or good results in 93% of the patients treated. The authors, based on data from a period of 5-year follow-up, concluded that treatment for DDH using this modified medial approach during early childhood is an effective and reliable method with low complication rates and that great success in radiologic and clinical outcomes could be expected [9]. Whether this finding could be extrapolated to older patients remains to be elucidated. This study presents a wide age range. In Iran, there are numerous neglected cases of DDH who present to the pediatric orthopedist very late. The fact that capsulorrhaphy could help the deep sitting of the femoral head even in these advanced cases is an argument that could be made in favor of capsulorrhaphy (Fig. 3). Currently, it is recommended that when the diagnosis has been made late, reduction of the hip must be combined with capsulorrhaphy along with corrective procedures on the femur and acetabulum to stabilize the reduction, with the goal of optimizing hip stability and minimizing the risk of residual dysplasia [12]. In the current study, the favorable effect observed on the acetabular-head contact, as indicated by deep-seating of the femoral head, due to capsulorrhaphy resisted adjustment for the confounding effect of age. This finding implies that the favorable effect of capsulorrhaphy on improving acetabular-head contact is independent of age.

The surgeons who support capsulorrhaphy would argue that it helps obtain and maintain a acetabular-head contact. Other surgeons, however, that recommend against capsulorrhaphy believe that not only is the capsulorrhaphy ineffective in obtaining and maintaining reduction, but also it may put the hip joint in jeopardy in terms of range of motion [29].

The Salter-type capsulorrhaphy is performed by excision of the redundant superolateral and posterior pouch of capsule to minimize the chance of re-dislocation. Proper excision and repair leaves no space into which the head could dislocate or subluxate [30].

Capsulorrhaphy and capsulectomy were prospectively compared by Lejman. They investigated a sample of 39 patients with DDH. The surgical technique included innominate osteotomy, and femoral shortening and derotational osteotomy. During follow-up, avascular necrosis developed in one capsulorrhaphy patient. Postoperative re-dislocation was reported to occur in three capsulorrhaphy and no capsulectomy patients. Three capsulorrhaphy and three capsulectomy patients had acetabular dysplasia. Neither capsulorrhaphy nor capsulectomy were found to produce superior hip motion at the last examination. The authors were convinced based on these findings to continue capsulectomy in conjunction with open reduction for DDH [14]. In the current study no case of avascular necrosis occurred and the only patients who couldn’t achieve full hip motion, were known cases of arthrogryposis.

The findings of the current study need to be interpreted in light of the limitations and strengths. Regarding generalizability of the results there are serious limitations in this study. There are a number of limitations to this study. An argument could be made as to the effect of femoral shortening and/or derotational osteotomy on the reported decrease in medial joint space. None of our patients underwent derotational osteotomy. Significant decrease was still apparent in the 13 patients who did not undergo femoral shortening osteotomy, nevertheless, in order to determine the exact effect, larger sample sizes would be needed. We reported no case of re-dislocation, instability, avascular necrosis or osteoarthritis. This absence of complications cannot be generalized due to the relatively low number of cases. The low sample size in this study is partly due to the fact that we only included unilateral DDH cases, so that we could compare postoperative range of motion to the contralateral side and determine if full motion had been restored.

A limitation of this study is the use of scalpels as magnification markers. In fact we used the Conn’s method of “placing a marker of known diameter beside the thigh at the level of the femur” [31]. Precise placement of external calibration markers (ECMs) is of paramount importance in this method [32]. A spherical marker would have been a more reliable choice. Nevertheless, in order to avoid measurement errors, extreme care was taken to maintain the patient, the C-arm and the marker in the exact same position and angle, so that the only difference between the two images was performing capsulorrhaphy.

Regarding study design the most powerful design would be to compare results in patients who have undergone capsulorrhaphy with those who haven’t. It might have been unethical not to perform capsulorrhaphy in a group of children. Howbeit, to overcome this flaw, we compared medial joint space before and after capsulorrhaphy to show its radiographic effect.

It is generally accepted that “the only guarantee of a lifetime of normal hip function is a completely normal radiographic appearance of the hip” [33]. Obtaining a completely normal radiographic appearance of the hip is a rationale, based upon which the treatment of DDH has been developed and still continues to develop. Herein, no claim can be made as to the clinical efficacy of capsulorrhaphy, as there were no control groups to compare re-dislocation rate and complications. Further long-term studies with larger sample sizes will be required before accepting the capsulorrhaphy as the sine qua non to the surgical treatment of the DDH. The strength of the current study lies in the statistical power that was large enough to enable us adjust in the effect of age. We used appropriate statistical method that is robust to the violation of the regression presumptions of normality on the distribution of the medial joint space measures as well as that of linearity in the association. When we log-transformed the measures to obtain more normally-distributed measures, the results remained essentially the same. We, therefore, decided to report the more parsimonious model without transformation.

## Conclusion

In conclusion, according to the current study capsulorrhaphy independently of age is associated with a decrease in the mean medial hip joint space and a more deeply seated femoral head. It seems that capsulorrhaphy is an effective technique in minimizing instability in the developmental dysplasia of the hip.

## Data Availability

The datasets used and/or analyzed during the current study are available from the corresponding author on reasonable request.

## Abbreviations

DDH: Developmental Dysplasia of the Hip
SD: standard deviation
ECM: external calibration marker
